# Place Cell Networks in Pre-weanling Rats Show Associative Memory Properties from the Onset of Exploratory Behavior

**DOI:** 10.1093/cercor/bhw174

**Published:** 2016-07-25

**Authors:** L. Muessig, J. Hauser, T. J. Wills, F. Cacucci

**Affiliations:** 1 Department of Neuroscience, Physiology and Pharmacology; 2 Department of Cell and Developmental Biology, University College London, London, UK

**Keywords:** development, hippocampus, memory, place cell

## Abstract

Place cells are hippocampal pyramidal cells that are active when an animal visits a restricted area of the environment, and collectively their activity constitutes a neural representation of space. Place cell populations in the adult rat hippocampus display fundamental properties consistent with an associative memory network: the ability to 1) generate new and distinct spatial firing patterns when encountering novel spatial contexts or changes in sensory input (“remapping”) and 2) reinstate previously stored firing patterns when encountering a familiar context, including on the basis of an incomplete/degraded set of sensory cues (“pattern completion”). To date, it is unknown when these spatial memory responses emerge during brain development. Here, we show that, from the age of first exploration (postnatal day 16) onwards, place cell populations already exhibit these key features: they generate new representations upon exposure to a novel context and can reactivate familiar representations on the basis of an incomplete set of sensory cues. These results demonstrate that, as early as exploratory behaviors emerge, and despite the absence of an adult-like grid cell network, the developing hippocampus processes incoming sensory information as an associative memory network.

## Introduction

The hippocampus has an essential role in the encoding of long-term memories, including spatial (Morris et al. 1982) and episodic memories (Scoville and Milner 1957). It contains “place cells”, neurons that fire only when an animal visits a restricted area of the environment (the “place field”). Their collective activity is thought to constitute a cognitive map of space (O'Keefe and Nadel 1978), which may also be combined with other aspects of experience (Tsao et al. 2013; Eichenbaum 2014; Knierim et al. 2014) to support more general associative memory (Marr 1971; Squire and Zola-Morgan 1991; Cohen and Eichenbaum 1995; Nadel and Moscovitch 1997; Eichenbaum 2004).

Hippocampal involvement in associative memory is thought to rely on the dense network of recurrent collaterals in the CA3 subfield, which could serve as an auto-associative network capable of pattern completion (Marr 1971). Pattern completion refers to the recall of a complete memory or neural representation following the presentation of a partial or degraded stimulus, allowing content addressable memory and accurate recall in the face of noisy input (Gardner-Medwin 1976; Hopfield 1982; McNaughton and Morris 1987; Treves and Rolls 1994; McClelland et al. 1995). Another key characteristic of an efficient associative memory network is pattern separation: the decorrelation of similar sensory inputs reaching the network, such as to minimize the overlap between their neural representations. Pattern completion without accompanying pattern separation results in a network highly prone to interference (McClelland et al. 1995). The end result of a network which combines pattern completion and separation is one in which neural activity represents a step function or sigmoid transformation of sensory input: gradually increasing the deviation from a familiar stimulus results in little change to the neural representation at first, then, after a certain threshold, a drastic switch to a new representation occurs. The key signature of pattern separation and completion is therefore a deviation from a linear transformation of inputs. (for a recent review of experimental data, see Yassa and Stark 2011).

Upon exposure to distinct environments, hippocampal place cells can display “remapping”, a change in either their firing rate (“rate remapping”) or both their firing rate and place field location (“global remapping”) (Muller and Kubie 1987; Bostock et al. 1991; Muller 1996; Leutgeb et al. 2005). Place cell remapping can be used to study both pattern separation and completion: if introducing animals to environments sharing a degree of similarity results in place cell remapping, this can be characterized as pattern separation; on the other hand, if the same manipulation results in unchanged place maps, this can be characterized as pattern completion.

Early studies measuring the responses of place cells to manipulations of sensory cues (O'Keefe and Conway 1978; Muller and Kubie 1987) showed that place cell maps are resistant to the removal of a partial subset of cues, but that they do change dramatically (displaying global remapping) following more drastic changes to the recording environment, consistent with pattern separation/completion in the place cell network. The strongest demonstration of pattern completion/separation in place cells, though, has come from studies in which environmental stimuli are varied incrementally within an experiment (Lee et al. 2004; Vazdarjanova and Guzowski 2004; Wills et al. 2005; Leutgeb and Leutgeb 2007; Neunuebel and Knierim 2014), allowing direct observation of nonlinear place cell responses (with the strength of such nonlinear responses differing between different hippocampal subfields; Lee et al. 2004; Leutgeb et al. 2004; Vazdarjanova and Guzowski 2004; Leutgeb and Leutgeb 2007; Neunuebel and Knierim 2014).

In this study, we tested when, during the postnatal development of rats, place cell maps show nonlinear responses to environmental modification, thus displaying behavior consistent with an associative memory network.

Place cells are present as early as postnatal day 16 (P16) (Langston et al. 2010; Wills et al. 2010) as soon as spontaneous exploration of the environment begins (Gerrish and Alberts 1996). However, it remains unknown whether place cells can encode and discriminate between multiple spatial contexts at such young ages, and by which age recall following partial cue presentation can be observed. Of particular interest is whether associative responses are present before weaning (P21): theoretical models have proposed that place cell remapping is initiated by grid cell input from the medial entorhinal cortex (mEC) (Monaco et al. 2011). However, adult-like grid cells have not been recorded before weaning age (Langston et al. 2010; Wills et al. 2010; Bjerknes et al. 2014). It is therefore of interest to test whether remapping can already occur before weaning, when stable and regular grid cell firing is still absent.

## Materials and Methods

### Subjects

Thirty-two male Lister-Hooded rat pups, aged P12–P22 and weighing 24–64 g on the day of surgery, were used as subjects. Litters were bred in-house and remained with their dams until weaning (P21). Rats were maintained on a 12:12 h light:dark schedule (lights off at 12:00). At P4, litters were culled to 8 pups/dam to minimize inter-litter variability. After surgery, each pup was separated from their mother for 30 min–3 h per day, to allow for electrophysiological recordings. For further details of the numbers of recording sessions run and cells recorded, see Supplementary Table 4*D*. Ten male Lister-Hooded adult rats, aged 4–6 months at the time of recording, were used as an adult control group.

### Surgery and Electrode Implantation

Rats were chronically implanted with microdrives loaded with 4–8 tetrodes, aimed at the hippocampal CA1 region (2.9 or 4.0 mm posterior to bregma and 1.8 or 2.5 mm lateral to bregma, in rat pups and adults, respectively). Tetrode position was confirmed by postmortem Nissl staining (see Supplementary Fig. 9).

### Single-Unit Recording

Rats were allowed a 1-day postoperative recovery, after which electrodes were initially advanced by 62–250 µm/day, but were moved in smaller increments (typically 62 µm) as the CA1 layer was approached (as determined by increasing amplitudes of ripple and theta-band oscillations), until the CA1 pyramidal layer was identified by the presence of complex-spike cells and 200 Hz “ripple” fast oscillations. Isolation of single units from multi-unit data was performed manually on the basis of peak-to-trough amplitude, using the software package “TINT” (Axona, Herts, UK); isolated units were required to show a 2 ms refractory period, as assessed by visual inspection of the temporal autocorrelogram. Environmental manipulations were not performed unless stable tetrode recordings (as assessed by the stability of extracellular waveforms) were observed across 2 or 3 trials in the familiar environment. Isolated single units were only used for further analysis if they fired ≥100 spikes in a given trial. Single units recorded in the CA1 were classified into complex-spike cells (putative pyramidal cells) and putative interneurons using *k*-means clustering, based on the following parameters: 1) spike width (peak-to-trough), 2) first moment of the temporal autocorrelogram within a 50-ms window, and 3) mean firing rate of the cell (Csicsvari et al. 1999).

### Recording Environments

The “familiar” environment consisted of a square, light-gray wooden box (walls 62.5 cm long, 50 cm high), placed on a smooth black plastic platform. Olfactory cues were never intentionally removed/altered from the familiar enclosure: the environment was not washed between trials (though fecal boli were removed and urine puddles were absorbed by paper towel, without being spread around). The odor traces that were left by the rats therefore accumulated across recording sessions. The “novel” environment consisted of a brown square plastic box (walls 61 cm long, 50 cm high) placed on an opaque platform located in a different location of the recording room and surrounded by black curtains, ensuring that no extramaze cues were shared between the familiar and novel environments. For all “visually identical replica” conditions (“rEnv”, “rFloor”, “rWalls”), the respective parts were exchanged for replicas of the same appearance made of the same material. These environments were placed in the same location as the familiar environment. In contrast to the familiar environments, the replacement walls and floor were washed between trials; hence, they lacked a clear set of accumulated odor cues. Between recording trials, rats were placed in a holding box (walls 20 cm long, 30 cm high), with sawdust and a heat pad on the floor. The holding box shared visual extramaze cues with the familiar environment but was screened by the black curtains from the novel environment.

### Behavioral Testing Protocol

**Figure 1.**
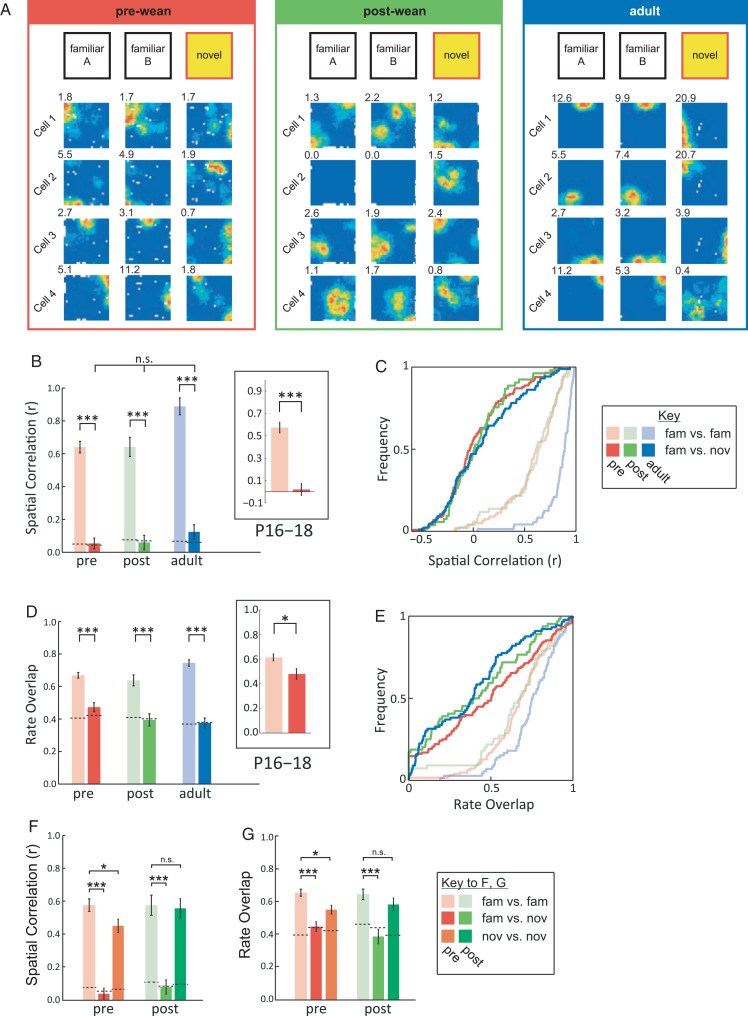
The place cells of pre-weanling rats undergo global remapping. (*A*) Examples of place cell ensembles in familiar and novel environment across development. Rate maps for 2 consecutive trials in the familiar environment (“familiar A” and “familiar B”, left) and 1 trial in the novel environment (“novel”, right). Peak firing rates are shown top left of each rate map (Hz). (*B*) Average spatial correlation (mean ± SEM) across familiar (pale colors) and familiar versus novel environments (bold colors) at different ages (red: pre-wean; green: post-wean; blue: adult). Dotted lines indicate chance levels. Inset shows data for animals aged P16–P18. (*C*) Same data as *B*, but shown as cumulative distribution functions (CDFs). (*D*) Average firing rate overlap (mean ± SEM) across familiar (pale colors) and familiar novel environments (bold colors) at different ages. (*E*) Same data as *D*, but shown as CDFs. ***P < 0.001, *P < 0.05. (*F,G*) Average spatial correlation (*F*) and rate overlap (*G*) (mean ± SEM) for those pre- and post-weanling animals exposed to the novel environment for 2 consecutive sessions. Dotted lines indicate chance levels. Average place cell stability within the novel environment is shown in orange (pre-weanling) and dark green (post-weanling), other colors as for *B–E*.

Rats were exposed to the familiar environment for a minimum of 5 trials (median across rats = 9 trials), over at least 2 days prior to the first exposure to manipulated environments (see Supplementary Fig. 5 for average exposure time to the familiar environment for pre- and post-weanling animals, for each environmental manipulation). On each experimental day, recording sessions began with 2–3 trials in the familiar environment. Animals would then be exposed to 1–3 different environmental manipulation trials, each for 1 trial only, and each manipulation was interleaved by a single trial in the familiar environment. An exception to this rule were those rats that received 2 consecutive recording trials in the novel environment (Fig. 1*F–G*). Rats were returned to the holding box for a 10–15 min inter-trial interval between all trials, including between the 2 consecutive trials in the novel environment. Supplementary Table 4*D* includes information regarding the age of animals, the number of cells recorded and sessions performed for each environmental manipulation, and age group. The precise number of recording trials run on each day depended on position sampling behavior of the rat: whenever position sampling was inadequate (defined as path length <45 m), data from that trial were discarded, and the experiment was stopped for the day (see Supplementary Fig. 7 for examples of dwell maps, showing age mean, and worst cases of environmental sampling for all age groups). Data included were obtained from both the first exposure to any given environment (for each rat), as well as repeat exposures. There were no significant differences in remapping between first and repeat exposures (see Supplementary Table 4 for further details). With the exception of rats being deliberately exposed to 2 consecutive trials of the novel environment (Fig. 1*F–G*), rats were not exposed to the same environment twice in the same day. In all recording trials, rats searched for drops of soya-based infant formula milk randomly scattered in the environment. Trials were 10–20 min long.

### Construction of Firing Rate Maps

Firing rat maps were constructed as described previously (Muessig et al. 2015). Briefly, positional data were sorted into 2.5 × 2.5 cm bins, spike and positional data were immobility-filtered (speed <2.5 cm/s for pups, <5 cm/s for adults), and the binned data smoothed using an adaptive smoothing algorithm (Skaggs et al. 1996).

### Criteria for Classification of Place Cells

As described previously (Muessig et al. 2015), complex-spike cells were classified as place cells if their spatial information scores (Skaggs et al. 1996) exceeded a threshold defined as the 95th percentile of a population of spatial information scores derived from age-matched, spatially shuffled data.

### Quantification of Remapping

Changes in the location of firing fields were quantified using the correlation (Pearson's *r*) between firing rate values of spatially corresponding bins in the rate maps of 2 trials, referred to here as “spatial correlation” (“SC”). Changes in place cell firing rate were quantified using rate overlap (“RO”; Leutgeb et al. 2004), defined as the ratio between the lower and the greater of the mean rates in the 2 trials being compared.

### Statistical Analysis

The inter-trial stability of place cells in a familiar spatial context changes with age (Langston et al. 2010; Wills et al. 2010); therefore, for each age group and for each different test of remapping, we first defined the baseline stability of place fields in the “familiar” environment, as the average of the inter-trial comparisons (SC and RO) between the first 2–3 “familiar” trials of each session (before the rat was exposed to any altered environments). Then, for each environmental manipulation, the degree of remapping was defined as the comparison (SC or RO) between the environmental manipulation trial and the temporally preceding “familiar” trial. To assess whether an environmental manipulation caused significant remapping, taking into account the developmental changes in baseline stability, the degree of remapping was tested using a 2 × 3 ANOVA, with an Environment factor of 2 levels (Baseline, Manipulation) and an Age factor of 3 (Pre-weanling, Post-weanling, Adult). If the ANOVA interaction term was significant, the difference between Baseline and Manipulation stability within each Age level was tested using simple main effects (SME). Full ANOVA results are reported in Supplementary Table 4*A*. The *P* values reported in the text refer to the main effect of Environment (when describing a remapping effect occurring at all ages), the Environment × Age interaction term (when describing a remapping effect differing across age groups), and the SME significance (when describing a remapping effect at one Age level in particular). SC and RO were treated equivalently at all stages, with the exception that SC Pearson's *r* values were transformed to Fisher's *Z* for the purposes of the ANOVA. For further confirmation of the results provided by ANOVAs, we also calculated the (uncorrected) *t*-tests between baseline and manipulation, for each age group and manipulation type (see Supplementary Table 4*B*).

In addition, the differences between distributions of SC and RO scores were tested using the Kolmogorov–Smirnov test (see Supplementary Table 4*C*), and remapping specifically within the P16–P18 age group was tested using *t*-tests.

In Figure 1 and Supplementary Figure 1, chance levels for correlations and RO were calculated by shuffling cell identities (separately for each age group) for a given manipulation trial and obtaining the age-matched average. This procedure was repeated 10 000 times to yield a distribution, of which the 95th percentile was used for defining the chance level.

All data analyses were performed with custom-written analysis tools using Matlab (MathWorks, Natick, USA), and all statistical analyses were conducted with SPSS (IBM Corporation, Armonk, USA).

## Results

### Remapping Occurs as Early as P16

To test whether pre-weanling place cells undergo remapping, we exposed rats to an environment comprised of a completely novel set of intra- and extra-maze cues (exposure to this magnitude of environmental changes induces “global remapping” in adult rats; Leutgeb et al. 2005). Place cells undergo global remapping at all ages: place fields shift to new positions or cease firing, while others, which were silent before, become active (example cells are shown in Fig. 1*A*; Supplementary Fig. 1*A–C* shows the full ensembles of co-recorded place cells from which these examples were drawn). We quantified changes in field position using SC and in firing rate using RO (Leutgeb et al. 2004). Comparing baseline levels of stability (Fig. 1*B*,*C*) and RO (Fig. 1*D*,*E*) in the familiar environment (pale colors) with those across familiar and novel environments (bold colors), we found that the hippocampus generates orthogonal place codes for the two environments throughout development, as both SC and RO measures approach chance levels for familiar-novel comparison at all ages (Fig. 1*B–E*; SC, *F*_1,496_ = 533.6, *P* < 0.001; RO, *F*_1,535_ = 130.4, *P* < 0.001; see Supplementary Table 4 for full statistical analysis). This is true even for the youngest rats, P16–P18 (see insets Fig. 1*B*,*D*; SC, *t*_(91)_ = 8.35, *P* < 0.001; RO, *t*_(101)_ = 2.56, *P* = 0.012). Moreover, when rats return to the familiar environment, the original representation is reinstated (see Supplementary Fig. S1*D–E*, gray bars), showing that different hippocampal representations co-exist independently of each other. To test whether place cell maps are stable in *both* familiar and novel environments, a subset of pre- and post-weanling rats were exposed to the novel environment for two consecutive sessions, separated by a 15-min interval: these data show that novel environment representations (data are shown as orange/dark green bars in Fig. 1*F–G*) are more stable than expected by chance in both age groups (compare orange/dark green bars with bold red/light green bars), notwithstanding a small decrease in stability with respect to the familiar environment (compare orange/dark green bars with pale red/light green bars), in pre-weanling animals only (Fig. 1*F–G*; see Supplementary Fig. S1*F–G* for example rate maps).

### Pre-weanling Place Cells Remap upon Changes to Local Olfactory Cues

**Figure 2.**
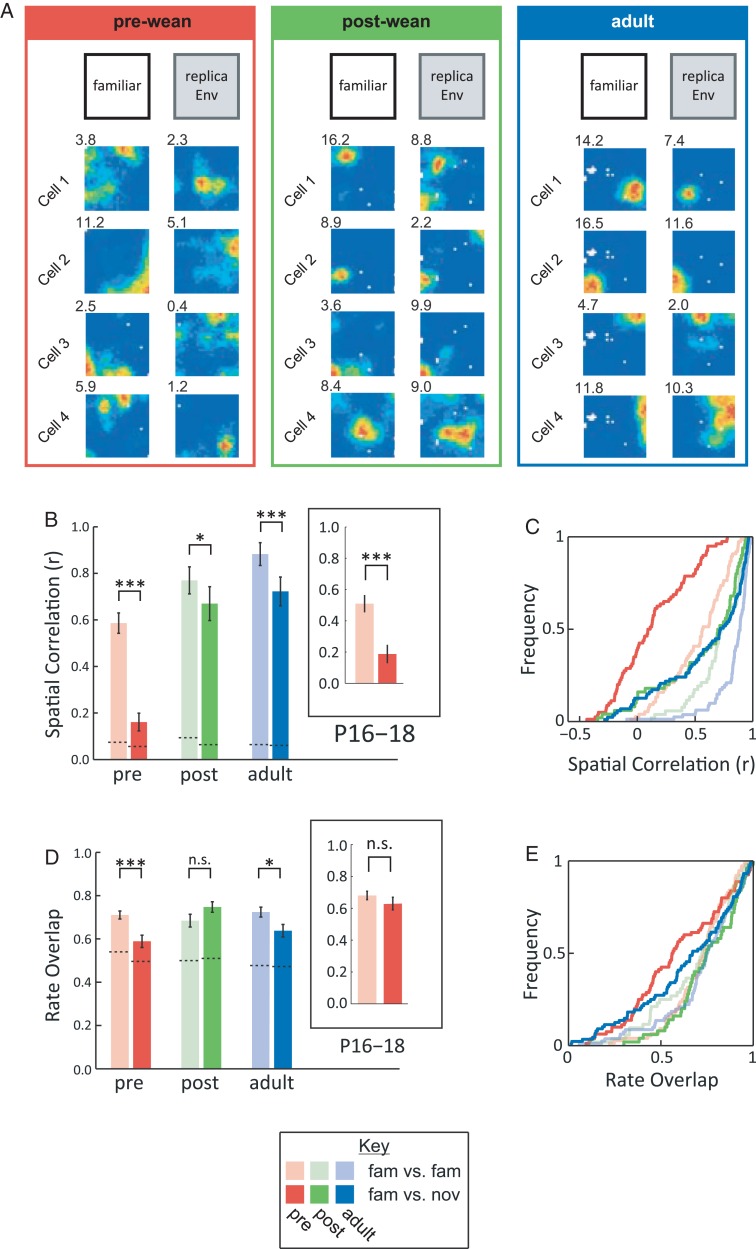
Pre-weanling place cells undergo remapping upon changes to local olfactory cues (*A*) Examples of place cell ensembles in familiar environment (“familiar”, left) and where the environment is replaced with a visually identical replica (“rEnv”, right), at different ages. (*B*) Average spatial correlation (mean ± SEM) across familiar trials (pale colors) and familiar versus “rEnv” trials (bold colors) at different ages (same colors as in Fig. 1). Dotted lines indicate chance levels. Inset shows data for animals aged P16–18. (*C*) Same data as *B*, but shown as CDFs. (*D*) Average firing rate overlap (mean ± SEM) across familiar trials (pale colors) and familiar “rEnv” comparison (bold colors) for different age groups. (*E*) Same data as *D*, but shown as CDFs. n.s., *P* ≥ 0.05; *P < 0.05; ***P < 0.001.

Global remapping follows changes to all intra- and extramaze cues. To investigate pattern separation in pre-weanling place cells, we exposed animals to a visually identical replica of the familiar environment (“rEnv”). This environment shares visual cues and environmental geometry with the familiar environment, while any intramaze olfactory cues that would have accumulated over repeated recording sessions are removed (see Materials and Methods). The “rEnv”, therefore, contains a strong degree of overlap with the familiar environment. We predicted that this manipulation might nevertheless produce strong remapping in pre-weanling rats in particular, due to the precocious development of the olfactory modality in mammals (Alberts 1984). Exposure to “rEnv” triggers some remapping at all ages (Fig. 2; see Supplementary Fig. 2; SC, *F*_1,420_ = 65.4, *P* < 0.001; RO, *F*_1,421_ = 4.83, *P* = 0.028), and, as predicted, a significantly greater degree of remapping is observed in pre-weanling rats, compared with post-weanling and adult rats (SC, *F*_2,420_ = 3.19, *P* = 0.042; RO, *F*_2,421_ = 5.73, *P* = 0.003; see Supplementary Table 4). Interestingly, “rEnv” triggers a specific remapping response in the subgroup of the youngest pre-weanling animals (P16–P18): place fields shift locations, but there are no significant changes in firing rate (see inset boxes in Fig. 2*B*,*D*; SC, *t*_(79)_ = 4.78, *P* < 0.001; RO, *t*_(79)_ = 1.06, *P* = 0.29). These results are consistent with the view that the pre-weanling hippocampus can orthogonalize overlapping input and generate distinct maps of environments (“rEnv” and “Familiar” environments) sharing a large degree of sensory similarity.

### Pattern Completion Occurs as Early as P16 in the rat

To establish whether early place cell maps display pattern completion, we then exposed rats to environments where either only the floor (“rFloor”) or only the walls (“rWalls”) were replaced with replicas (as contrasted to “rEnv”, where both the walls and floor were replaced). This experimental design is analogous to studies that set out to study pattern completion in place cells by comparing the effects of full and partial cue presentation on place cell firing (O'Keefe and Conway 1978; Nakazawa et al. 2002; Nakashiba et al. 2012).

**Figure 3.**
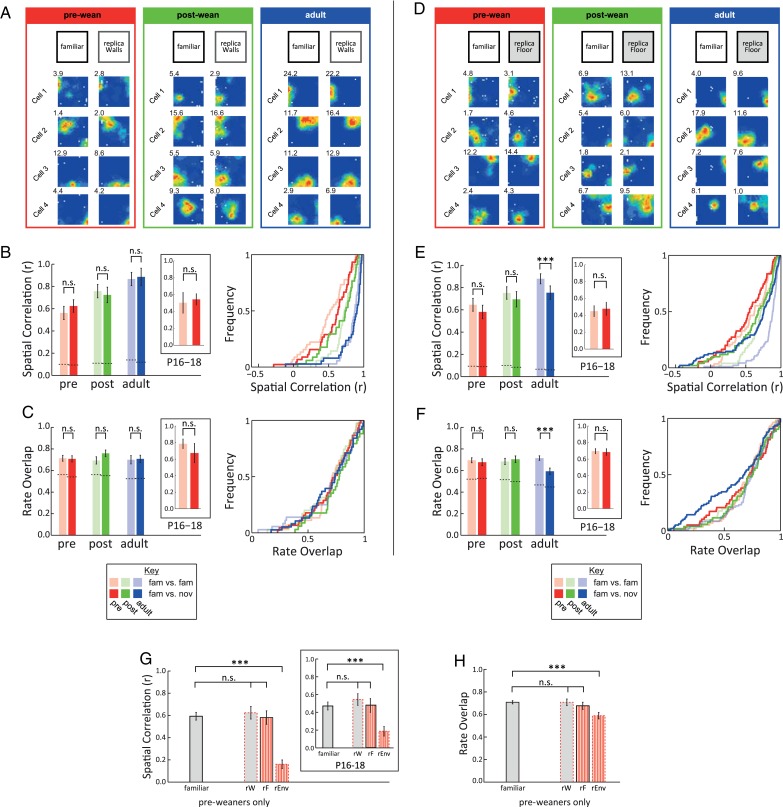
Pattern completion occurs in pre-weanling place cells. (*A–C*) Replacing the environment walls for a visually identical replica (“rWalls”) does not affect place maps. (*A*) Examples of place cell ensembles in familiar environment (familiar, left) and in the “rWalls” condition (“rWalls”, right), at different ages. (*B*) Average spatial correlation (mean ± SEM, left panel; CDFs, right panel) across familiar trials (pale colors) and familiar versus “rWalls” condition (bold colors), at different age groups (colors as in Fig. 1). Dotted lines indicate chance levels. Inset shows data for animals aged P16–18. (*C*) Average firing rate overlap (mean ± SEM, left panel; CDFs, right panel) across familiar trials (pale colors) and familiar versus “rWalls” condition (bold colors), at different ages. (*D–F*) Replacing the environment floor for a visually identical replica (“rFloor”) only affects place maps in adult rats. (*D*) Examples of place cell ensembles in familiar environment (familiar, left) and in the “rFloor” condition (“rFloor”, right), at different ages. (*E–F*) as in B and C but for familiar and “rFloor” conditions. (*G–F*) Pattern completion is already functional before weaning. (*G*) Spatial correlations (mean ± SEM) between familiar trials and the 3 trials with environmental manipulations of the local non-visual intramaze cues (“rEnv”, “rFloor”, “rWalls”) for pre-weanling animals only. Inset shows data for animals aged P16–18. (*H*) Same as *G*, but for firing rate overlap. n.s., *P* ≥ 0.05, ***P < 0.001.

The “rWalls” environment does not induce significant changes in place cell firing as assessed by SC and RO, in any age group (Fig. 3*A–C*; SC, *F*_1,227_ = 0.39, *P* = 0.53; RO, *F*_1,227_ = 0.76, *P* = 0.39; see Supplementary Fig. 3*A–E* for complete ensembles; see Supplementary Table 4 for statistical analysis). Note that baseline levels (pale bars/CDFs) are almost identical to familiar probe comparisons (bold bars/CDFs) for both measures in every age group.

The “rFloor” environment also has no significant effect on pre- and post-weanling place cells (Fig. 3*D–F*; SC: Age × Manipulation, *F*_2,400_ = 3.43, *P* = 0.033; post hoc: Pre: *P* = 0.30; Post: *P* = 0.67; RO: Age × Manipulation, *F*_2,406_ = 3.8, *P* = 0.023; post hoc: Pre: *P* = 0.67; Post: *P* = 0.65; see Supplementary Fig. 3*F–J* for complete ensembles), though a small but significant amount of remapping is observed in adults (post hoc tests for adults; SC, *P* < 0.001; RO, *P* < 0.001; see Supplementary Table 4).

Focusing specifically on the pre-weanling group, the results of “rEnv”, “rWalls”, and “rFloor” taken together (cf. Fig. 2 and Fig. 3*A–F*), show that pre-weanling place cell firing is consistent with pattern completion. When directly comparing the pre-weanling remapping scores for “Familiar”, “rEnv”, “rWalls”, and “rFloor” (Fig. 3*G–H*), we found that the “rEnv” manipulation results in significant remapping (SC: *F*_3,319_ = 31.06, *P* < 0.001; Tukey HSD “Familiar” vs. “rEnv”, *P* < 0.001; RO: *F*_3,322_ = 6.04, *P* = 0.001; Tukey HSD “Familiar” vs. “rEnv”, *P* < 0.001), demonstrating that intramaze olfactory cues are salient to the rat, and that they are required for place cell stability. However, neither “rFloor” nor “rWalls” has any effect on place cell stability (Tukey HSD *P* > 0.75 for “rWalls” and “rFloor” compared with familiar, for both SC and RO), showing that exposure to partial sets of familiar cues (either the walls or floor of the environment) is therefore sufficient to lead to the reactivation of the full place cell map. Interestingly, the same pattern completion effect is observed in the youngest animals (P16–P18), as shown by SC (inset Fig. 3*G*; *F*_3,128_ = 7.86, *P* < 0.001; Tukey HSD “Familiar” vs. “rEnv”, *P* < 0.001 vs. *P* > 0.75), despite the lack of significant rate changes at these ages (Fig. 2*D* inset).

Note that there was no systematic variation between “Familiar” and any type of environmental manipulation in either the amount of previous exposure to the familiar environment (see Supplementary Fig. 5) or general behavior as assessed by average running speed (see Supplementary Fig. 6) and positional sampling of the environments (see Supplementary Fig. 7), discounting these as potential confounds.

## Discussion

We have shown that place cells display adult-like remapping when animals are exposed to completely novel environments, already at P16, the earliest age at which rats engage in exploratory behaviors (Gerrish and Alberts 1996; Wills et al. 2014). This demonstrates that the hippocampus can generate and store multiple maps of different environments, as early as stable place cell firing has been documented (P16) (Langston et al. 2010; Wills et al. 2010). Importantly, remapping is triggered when animals are introduced into 1) completely different environments (the novel environment, with only geometry preserved between the novel and familiar environments, while all intra- and extramaze cues are novel) and 2) environments sharing a large set of common cues (“rEnv” and familiar environments where geometry and all visual cues, both intra- and extramaze, are preserved). Our conclusions primarily concern “global” remapping, as opposed to pure “rate” remapping (see Leutgeb et al. 2005 for distinction), as pure rate remapping is observed most clearly in the CA3 region in adult rats (Leutgeb et al. 2005), whereas the data in this study were collected from region CA1.

The presence of place cell remapping in the “rEnv” condition shows that the pre-weanling hippocampus can discriminate environments sharing a substantial degree of sensory similarity (“rEnv” and “Familiar” environments, sharing all visual cues) and encode them using independent, decorrelated neural codes, already at P16. These results are therefore consistent with the conclusion that hippocampal place maps already show pattern separation as soon as place responses can be recorded.

This study also offers the first demonstration of pattern completion in pre-weanling place cells. We tested pattern completion using the well-established cue removal experimental procedure (O'Keefe and Conway 1978; Nakazawa et al. 2002; Gold and Kesner 2005; Nakashiba et al. 2012).

Animals were introduced in environments in which either all local non-visual cues were changed (“rEnv”) or only partial, complementary, sub-sets of these cues were changed (“rFloor” and “rWalls”). We chose to manipulate the proximal olfactory cues as this sensory modality is the first to mature during mammalian development (Alberts 1984), and therefore, olfactory cues are likely to be the most salient cues available for spatial localization to pre-weanling rats. Place cell maps of pre-weanling rats remained unchanged in the “rFloor” and “rWalls” conditions, while complete remapping was observed in the “rEnv” condition, demonstrating that the hippocampus can recall maps of previously experienced (familiar) environments, upon exposure to only a subset of the familiar cues.

The key signature of pattern separation/completion is a nonlinear response to linear changes in input stimuli. In our results, such a nonlinear response is apparent when contrasting place cell responses in the “rEnv”, “rWalls”, and “rFloor” conditions (see Fig. 3*G*): a linear response would predict that the perturbation caused by “rFloor” and “rWalls” would sum to that of “rEnv”: instead, both “rFloor” and “rWalls” produce no effect, while “rEnv” elicits strong remapping. While the most stringent test of pattern separation and completion is to expose animals to a series of environmental manipulations where one variable is parametrically changed along a single dimension (geometry: Leutgeb et al. 2005, 2007; Wills et al. 2005; intra- and extra-maze cue mismatch: Lee et al. 2004), the number of recording trials required for this approach precludes its use in pre-weanling animals. Notwithstanding this technical limitation, when comparing the Novel, “rEnv” and familiar manipulations together, our results show that increasing environmental change (familiar < rEnv < Novel) leads to a nonlinear, all-or-none remapping response, thus supporting our interpretation that the developing hippocampus already displays pattern separation and completion at the earliest ages sampled (P16).

In summary, the results reported here are consistent with the hypothesis that the hippocampus is capable of associative encoding and recall as soon as rats engage in spatial exploration. These results might help inform the long-standing debate over when, during human development, children start displaying associative learning. In particular, we note here that there is some evidence for early associative capabilities in human infants as young as 6 months old (for a thorough review of this subject, see Mullally and Maguire 2014). Our study demonstrates that the mammalian hippocampus is capable of supporting associative learning in the spatial domain early during postnatal development, at a time when rats are just starting to display spatial exploration and, therefore, support the view that these capabilities should already be present in the human infants. We also note here that there is some evidence linking the onset of self-displacement (crawling in humans) with the emergence of flexible memory in human infants (Herbert et al. 2007), raising the possibility of an interesting parallel between rat and human development.

Our experiments were conducted in the CA1 region, a key output station for the entire hippocampal formation. The spatial selectivity of CA1 place cells, may, in turn, reflect spatial firing in two key input areas: CA3 and entorhinal cortex. The most prominent spatial signal in the entorhinal cortex are grid cells (Hafting et al. 2005), and the observation that grid maps realign in novel environments (Fyhn et al. 2007) inspired the hypothesis that grid cell realignment drives hippocampal remapping (Fyhn et al. 2007; Monaco et al. 2011). It is therefore notable that we find adult-like place cell remapping in pre-weaning animals, despite the lack of adult-like spatially stable grid cells at these ages (Langston et al. 2010; Wills et al. 2010; Bjerknes et al. 2014). These data conclusively rule out that the realignment of regular and stable grid cells is necessary for remapping, at least in developing rats, and confirm recent findings in adult rats (Brandon et al. 2014; Hales et al. 2014), suggesting that place cell remapping is grid cell independent also in adulthood. Although we cannot exclude that the immature, irregular spatial firing of putative grid cell precursors present in the EC before weaning (Derdikman and Moser 2010) might drive remapping, their firing patterns are extremely noisy (Langston et al. 2010; Wills et al. 2010), and therefore, it is unlikely that these could convey a coherent remapping signal to hippocampal place cells. We believe it more parsimonious to propose that remapping in the hippocampus does not require functional grid cell firing in pre-weanling rats.

What drives remapping in young pups remains an open question. It is also notable that the dentate gyrus, which is thought to be the ultimate driver of pattern separation in the hippocampus (Marr 1971; McNaughton and Morris 1987; Treves and Rolls 1994; Gilbert et al. 2001; Leutgeb et al. 2007), develops late, following a slower maturation than the CA fields where place cells are found (Bayer 1980).

Pattern completion in place cell networks is commonly held to be based on the recurrent connectivity of CA3 (Marr 1971). Our finding of pattern completion in young pre-weanling rats is therefore consistent with evidence from in vitro recordings that recurrent connectivity is already in place in CA3 by the second postnatal week (Gómez-Di Cesare et al. 1997). Here, we provide the first functional demonstration that CA1 place maps can perform pattern completion and therefore display critical features of associative networks, as soon as place cells can be recorded. An interesting contrast between remapping in pre-weanling and adult rats is that the pre-weanling CA1 place cells studied here showed a greater propensity for all-or-none remapping, compared with adults, where CA1 often shows partial or intermediate responses (Lee et al. 2004; Leutgeb et al. 2004). We speculate that, in the absence of mature entorhinal cortex input (see above), CA1 inputs may more faithfully reflect auto-associative inputs from CA3.

We note an interesting and possibly important dissociation between the early emergence of associative encoding in place cells, reported here, and development of place cell accuracy in a familiar environment, which we investigated in a previous experiment (Muessig et al. 2015). Muessig et al. (2015) showed that, before weaning, place cells are more accurate and reliable close to environmental boundaries than in the center of an open field environment. At weaning (and co-incidentally with the emergence of grid cells), place cells become equally accurate throughout space. This result suggests that grid cells may have a specific role in spatial cognition, allowing accurate navigation when an animal is far from environmental landmarks. The developmental dissociation between accurate, environment-wide mapping and associative memory encoding suggests that these processes are based on different neural substrates developing on distinct timescales.

We found that changing all intramaze olfactory cues (in “rEnv”) results in strong place cell remapping in pre-weanling rats, even though all visual (intra- and extramaze) cues remain constant. This result indicates that the sensory modalities supporting place cell firing mirror the general pattern of sensory development whereby the chemical senses (along with tactile sensation) develop first and vision latest. For example, rat pups can recognize the odor of their mother by P2 (Polan and Hofer 1999) and their home cage by P12 (Gregory and Pfaff 1971), whereas eyes remain closed until P14, and visual neural responses continue to mature for at least 2 weeks afterwards (Fagiolini et al. 1994; Prévost et al. 2010). The development of visual behavioral responses has been studied only in mice: but here also, eyes open at P15 and visual responses take approximately 2 weeks to mature (Prusky et al. 2004). Olfactory cues are also important for place cell stability in adult rats, if no visual cues are available (Save et al. 2000). We also note that, although the “rEnv” manipulation was primarily aimed at removing olfactory cues, we cannot rule out that very subtle changes in tactile cues (for example, changes in paint texture) may have also been detected by the rats. It is known that tactile cues can control place cell firing in adult rats (Gener et al. 2013). The increased dependence of pre-weanling place cells on boundaries (Muessig et al. 2015, see above) may also explain the seemingly paradoxical result that changing olfactory cues on the floor has a weaker effect in pre-weanling rats than in adults, despite olfactory cues appearing more important in general at this age. The increased weighing given to boundary cues may allow better compensation (via pattern completion) of the changed olfactory cues on the floor.

The presence of pattern completion in the pre-weanling hippocampus also suggests that, like in the adult, the earliest hippocampal place cells are integrating information from a constellation of cues and are therefore already coding for an abstract construct of space (O'Keefe and Conway 1978; Muller and Kubie 1987). As previous studies of pre-weanling place cells did not include any environmental manipulations (Langston et al. 2010; Wills et al. 2010; Muessig et al. 2015), the possibility remained that seemingly spatially selective firing was in reality driven by a single, spatially localized cue (e.g., an odor trace). We argue that our pattern completion results rule out that possibility, and in this sense, we have demonstrated here that spatially responsive CA1 pyramidal cells are truly “place cells,” even in the youngest rats. We accept the caveat, however, that we cannot conclude whether the conjunctions of cues supporting early place firing are multi-modal, as in the adult (O'Keefe and Conway 1978; Save et al. 2000) or based primarily on one sensory modality. In conclusion, our results suggest that the hippocampus processes incoming information in an obligatory associative fashion, and that this property is unlikely to require extensive experience-dependent remodeling of hippocampal connectivity after animals start to actively explore their environment.

## Supplementary Material

Supplementary material can be found at: http://www.cercor.oxfordjournals.org/.

## Funding

We acknowledge funding from the European Research Council (“DEVSPACE” grant to F.C.), the Biotechnology and Biological Sciences Research Council (grant BB/I021221/1 to F.C.), the Royal Society (URF fellowship to T.W.), and the Swiss National Science Foundation (fellowship to J.H.). Funding to pay the Open Access publication charges for this article was provided by University College London (see https://www.ucl.ac.uk/library/open-access).

## Supplementary Material

Supplementary Data
