# Supplementary material for: Place Cell Networks in Pre-weanling Rats Show Associative Memory Properties from the Onset of Exploratory Behavior
Source: Cereb Cortex. 2016 Jul 25;26(8):3627–36. doi: 10.1093/cercor/bhw174 (PMC4961032; doi:10.1093/cercor/bhw174)
Supplement: Supplementary Data [file supp_26_8_3627__index.html]

Place Cell Networks in Pre-weanling Rats Show Associative Memory Properties from the Onset of Exploratory Behavior — Place Cell Networks in Pre-weanling Rats Show Associative Memory Properties from the Onset of Exploratory Behavior — Supplementary Data 

# Place Cell Networks in Pre-weanling Rats Show Associative Memory Properties from the Onset of Exploratory Behavior

## Supplementary Data

Supplementary Data

- Supplementary Figure 1 - pdf file
- Supplementary Figure 2 - pdf file
- Supplementary Figure 3 - pdf file
- Supplementary Table 4 - pdf file
- Supplementary Figure 5 - pdf file
- Supplementary Figure 6 - pdf file
- Supplementary Figure 7 - pdf file
- Supplementary Figure 8 - pdf file
- Supplementary Figure 9 - pdf file
