# Supplementary material for: Place Cell Networks in Pre-weanling Rats Show Associative Memory Properties from the Onset of Exploratory Behavior
Source: Cereb Cortex. 2016 Jul 25;26(8):3627–36. doi: 10.1093/cercor/bhw174 (PMC4961032; doi:10.1093/cercor/bhw174)
Supplement: Supplementary Data [file supp_bhw174_bhw174supp_fig1.pdf]

Supplemental Figure 1

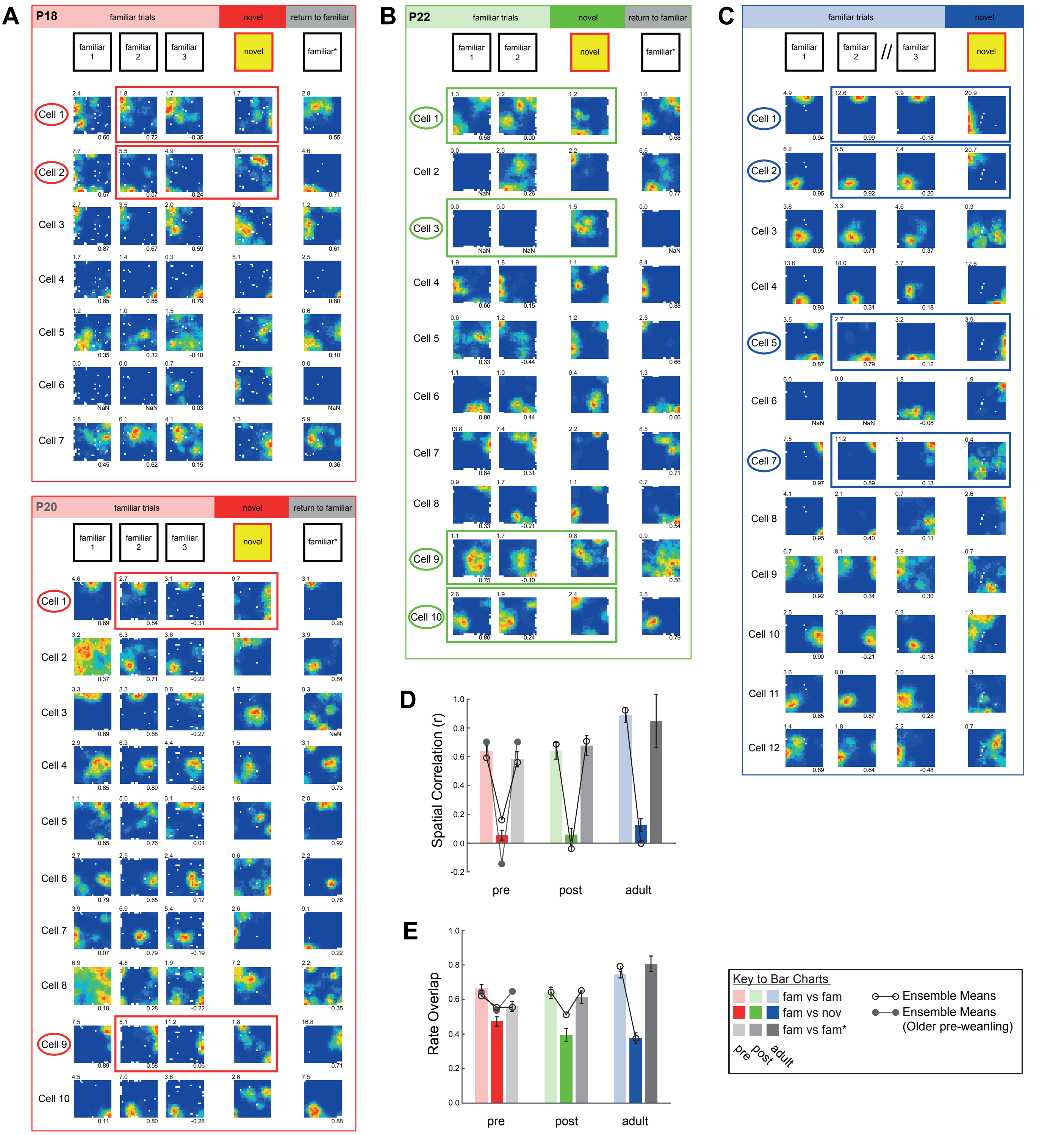

Supplemental Figure 1. Pre-weanling place cells generate independent and stable representations across familiar and novel environments.

(**A-C**) Complete ensembles of simultaneously recorded place cells from which examples in main Figure 1A are drawn:

(**A**) Pre-weanling rats (two separate ensembles, from a younger and an older animal); (**B**) Post-weanling rats and (**C**) adult animals. The cells in the familiar environment following the exposure to the novel environment is also shown, whenever available (“return-to-familiar”). The ‘//’ symbol between familiar trials indicates that another environmental manipulation (not shown in this figure) was run in between the sessions shown. The numbers on bottom right of rate maps indicate spatial correlation (r) with the following trial, except for ‘familiar*’ trials where the correlation with the preceding familiar trial is indicated.

(**D, E**) Overall age group means (±SEM) for spatial correlation (**D**) and rate overlap (**E**). These panels show the same data as main Figure 1B, 1D, but additionally show: the mean values of spatial correlation and rate overlap for the ensembles shown in S1A-C (black and grey lines and circles), and the overall mean (±SEM) comparisons between the familiar trials preceding and following the ‘novel’ trial (grey bars; ‘return-to-familiar’). There are no significant differences between familiar and return-to-familiar spatial correlation (ANOVA: Trial, $F_{1,369}$=0.66, p=0.42; Trial x Age, $F_{2,369}$=1.07, p=0.34) or rate overlap (ANOVA: Trial, $F_{1,384}$=0.39, p=0.53; Trial x Age, $F_{2,384}$=2.14, p=0.12).

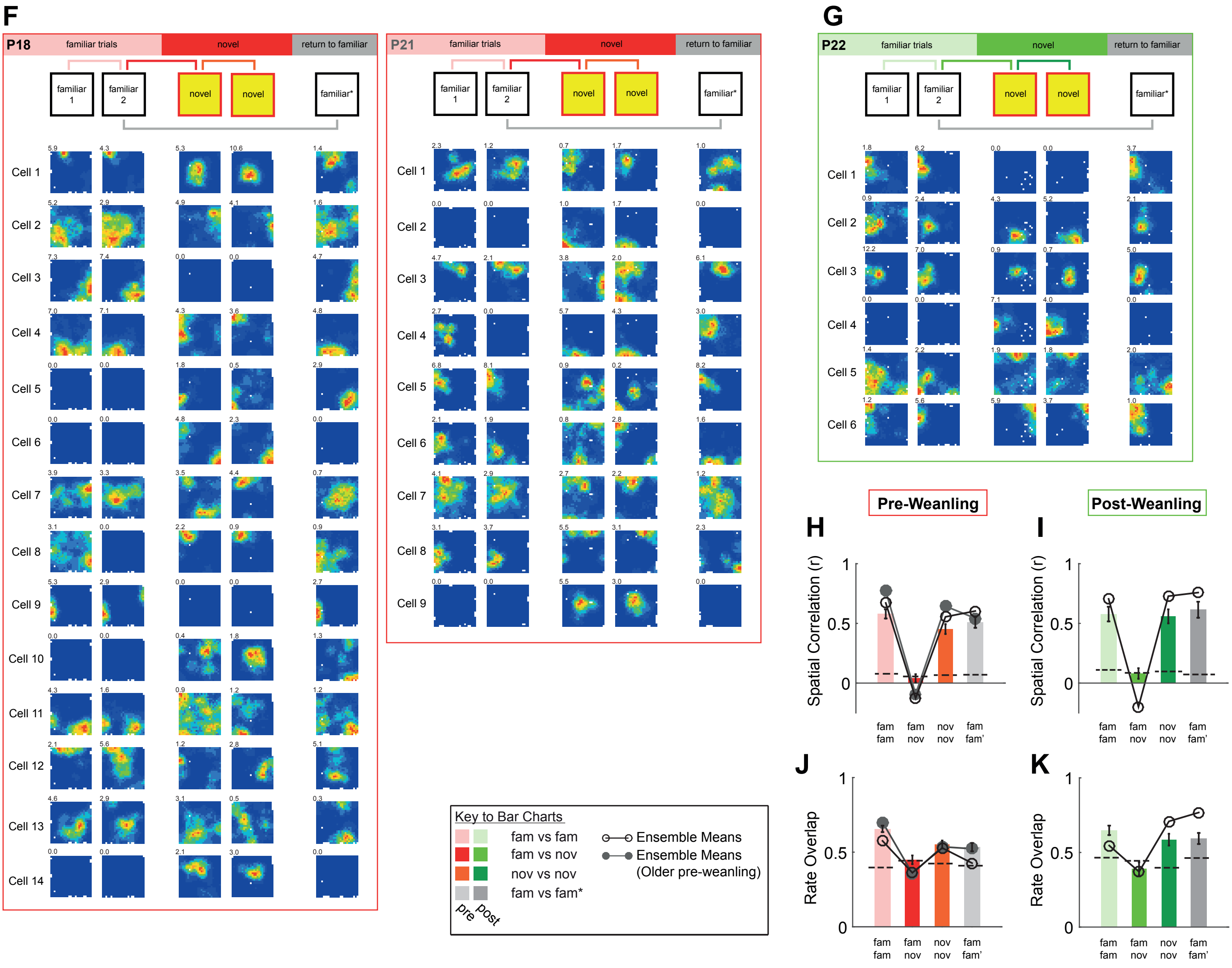

Supplemental Figure 1, continued.

(**F, G**) Complete ensembles of simultaneously recorded place cells where pre-weanling (**F**) and post-weanling (**G**) rats were exposed to the novel environment for two consecutive trials, showing that novel place representations are successfully recalled upon a repeated exposure to the novel environment.

(**H-K**) Overall age group means (±SEM) for spatial correlation (**H, I**) and rate overlap (**J, K**). These panels show the same data as main Figure 1F-G, but additionally show: the mean values of spatial correlation and rate overlap for the ensembles shown in S1F-G (black and grey lines and circles), and the overall mean (±SEM) comparisons between the familiar trials preceding and following the two 'novel' trial (grey bars; 'return-to-familiar'). Dashed lines across bars represent 95% chance levels, based on shuffled data (see Methods).
