# Supplementary material for: Place Cell Networks in Pre-weanling Rats Show Associative Memory Properties from the Onset of Exploratory Behavior
Source: Cereb Cortex. 2016 Jul 25;26(8):3627–36. doi: 10.1093/cercor/bhw174 (PMC4961032; doi:10.1093/cercor/bhw174)
Supplement: Supplementary Data [file supp_bhw174_bhw174supp_fig2.pdf]

Supplemental Figure 2

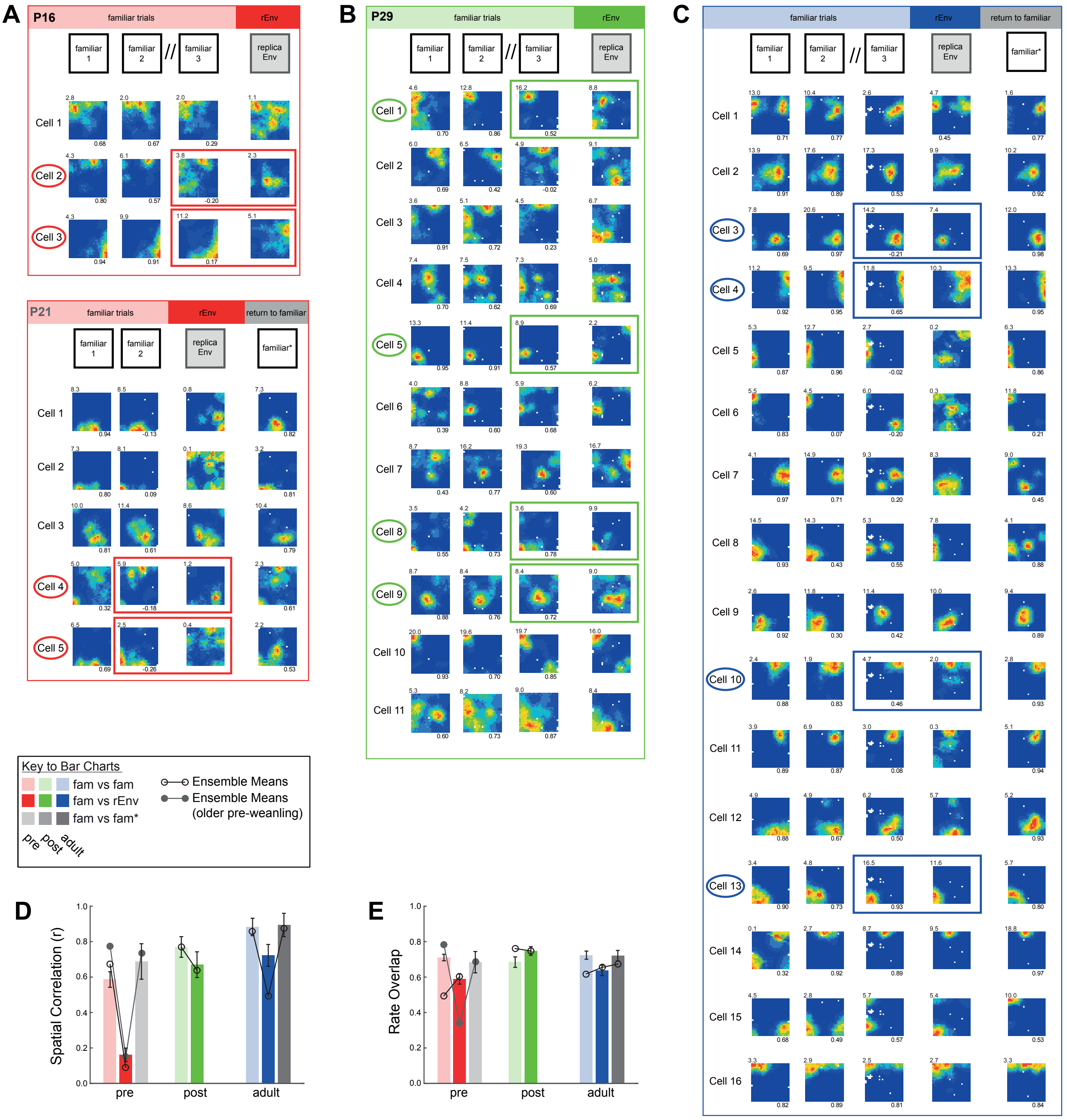

Supplemental Figure 2. Replacing the environment floor and walls with visually identical replicas results in remapping in all age groups, with the strongest effect observed in pre-weanling rats.

(**A-C**) Complete ensembles of simultaneously recorded place cells from which examples in main Figure 3A are drawn:

(**A**), pre-weanling rats (two separate ensembles, from a younger and an older animal); (**B**), post-weanling rats and (**C**), adult animals. The cells shown in main Figure 3A are highlighted with a circle around the cell number, to the left of the rate maps. Note that an additional trial in the familiar environment following the exposure to 'rEnv' is also shown whenever available ("return-to-familiar"). The '//' symbol between familiar trials indicates that another environmental manipulation (not shown in this figure) was run in between the sessions shown. The numbers on bottom right of rate maps indicate spatial correlation (r) with the following trial, except for 'familiar*' trials where the correlation with the preceding familiar trial is indicated.

(**D, E**) Overall age group (±SEM) for spatial correlation (**D**) and rate overlap (**E**). The panels show same data as main Figure 3B, 3D, but additionally show: the mean values of spatial correlation and rate overlap for the ensembles shown in S3A-C (black and grey lines and circles), and the overall mean (±SEM) comparisons between the familiar trials preceding and following the environmental manipulation (grey bars; 'return-to-familiar'). Note that no 'return-to-familiar' trials were available for post-weanling animals. There are no significant differences between familiar and return-to-familiar spatial correlation (ANOVA: Trial, $F_{1,217}$=1.57, p=0.21; Trial x Age, $F_{2,217}$=0.42, p=0.52), or rate overlap (ANOVA: Trial, $F_{1,218}$=0.15, p=0.70; Trial x Age, $F_{2,128}$=0.10, p=0.75).
