# Supplementary material for: Place Cell Networks in Pre-weanling Rats Show Associative Memory Properties from the Onset of Exploratory Behavior
Source: Cereb Cortex. 2016 Jul 25;26(8):3627–36. doi: 10.1093/cercor/bhw174 (PMC4961032; doi:10.1093/cercor/bhw174)
Supplement: Supplementary Data [file supp_bhw174_bhw174supp_fig3.pdf]

Supplemental Figure 3

Replica Walls

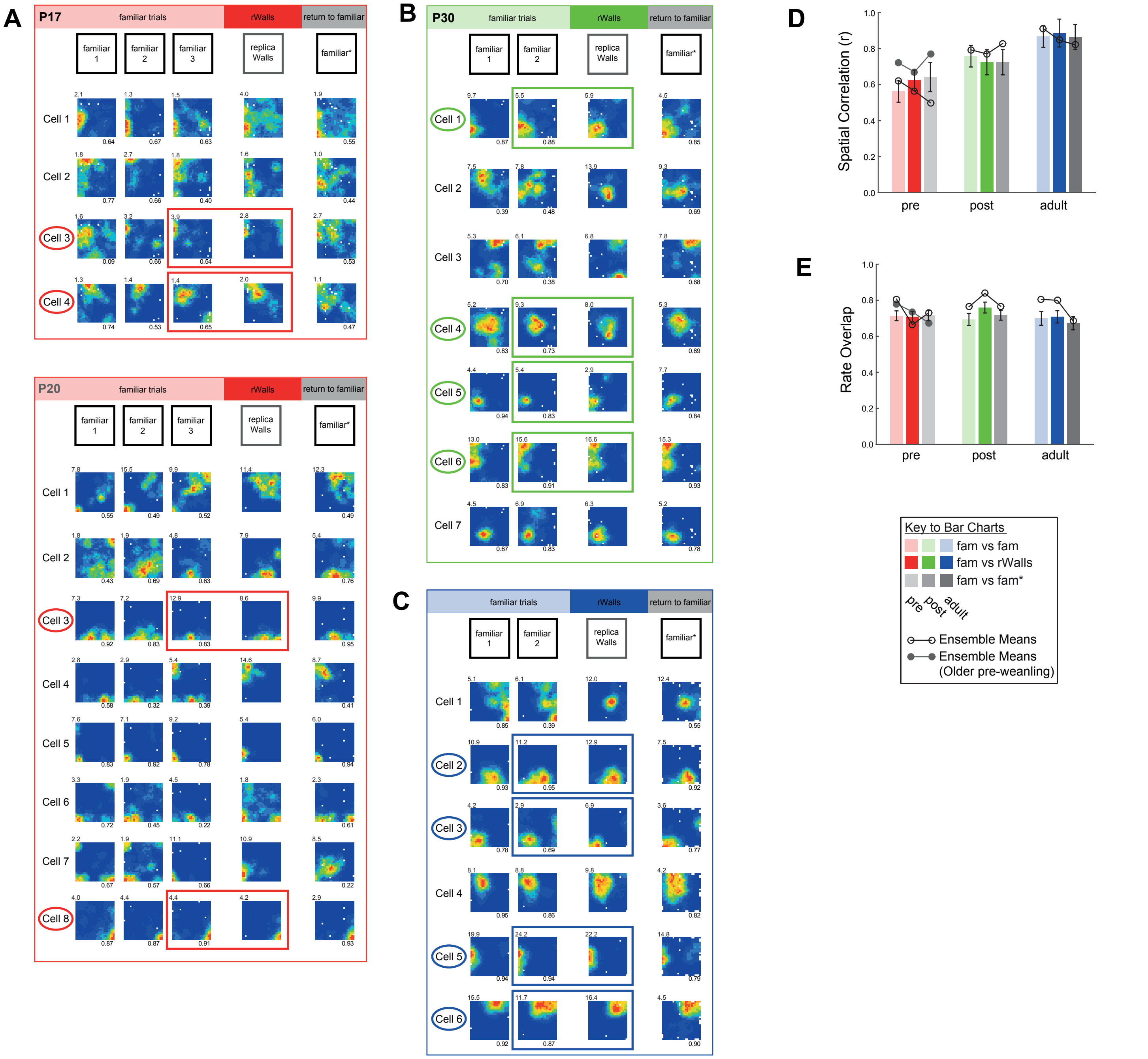

Supplemental Figure 3. Changing olfactory cues on either the walls or the floor of the recording arena does not disrupt pre-weanling place cells.

(**A-E**) ‘rWalls’ condition, where only the walls of the environment were replaced with a visually identical replica.

(**A-C**) Complete ensembles of simultaneously recorded place cells from which examples in main Figure 4A are drawn: (**A**), pre-weanling rats (two separate ensembles, from a younger and an older animal); (**B**), post-weanling rats and (**C**), adult animals. The cells shown in main Figure 4A are highlighted with a circle around the cell number, to the left of the rate maps. Note that an additional trial in the familiar environment following the exposure to ‘rWalls’ is also shown, whenever available (“return-to-familiar”). The numbers on bottom right of rate maps indicate spatial correlation (r) with the following trial, except for ‘familiar*’ trials where the correlation with the preceding familiar trial is indicated.

(**D, E**) Overall age group means (±SEM) for spatial correlation (**D**) and rate overlap (**E**). These panels show the same data as Figure 4B-C, but additionally show: the mean values of spatial correlation and rate overlap for the ensembles shown in S4A-C (black and grey lines and circles), and the overall mean (±SEM) comparisons between the familiar trials preceding and following the environmental manipulation (grey bars; ‘return-to-familiar’). There are no significant differences between familiar and return-to-familiar spatial correlation (ANOVA: Trial, $F_{1,215}=0.06$, $p=0.81$; Trial x Age, $F_{2,125}=1.04$, $p=0.36$), or rate overlap (ANOVA: Trial, $F_{1,215}<0.01$, $p=0.97$; Trial x Age, $F_{2,215}=0.30$, $p=0.74$).

Replica Floor

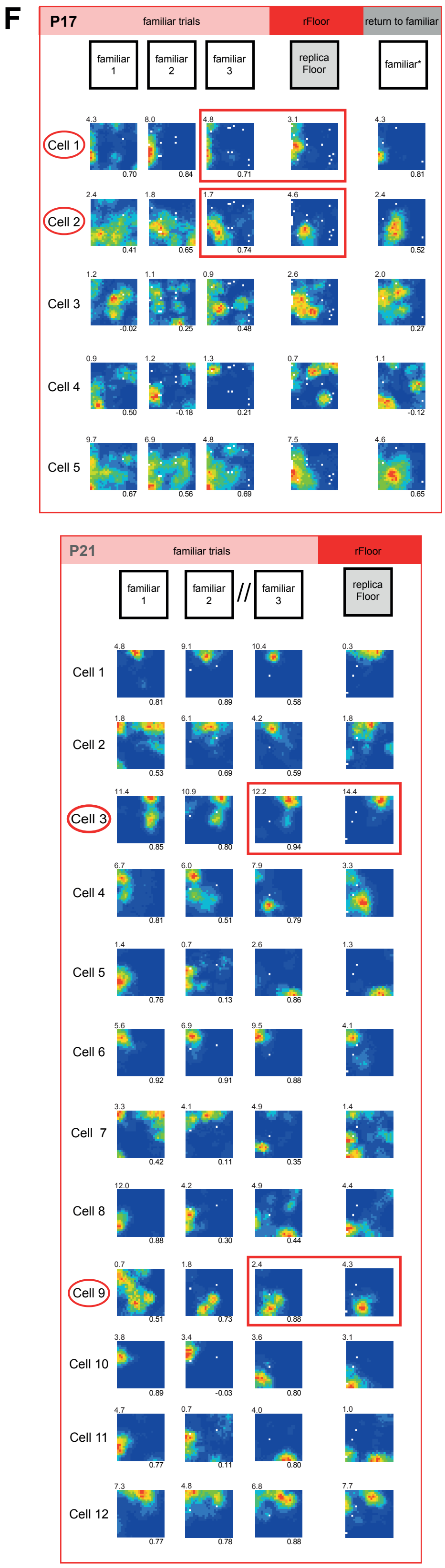

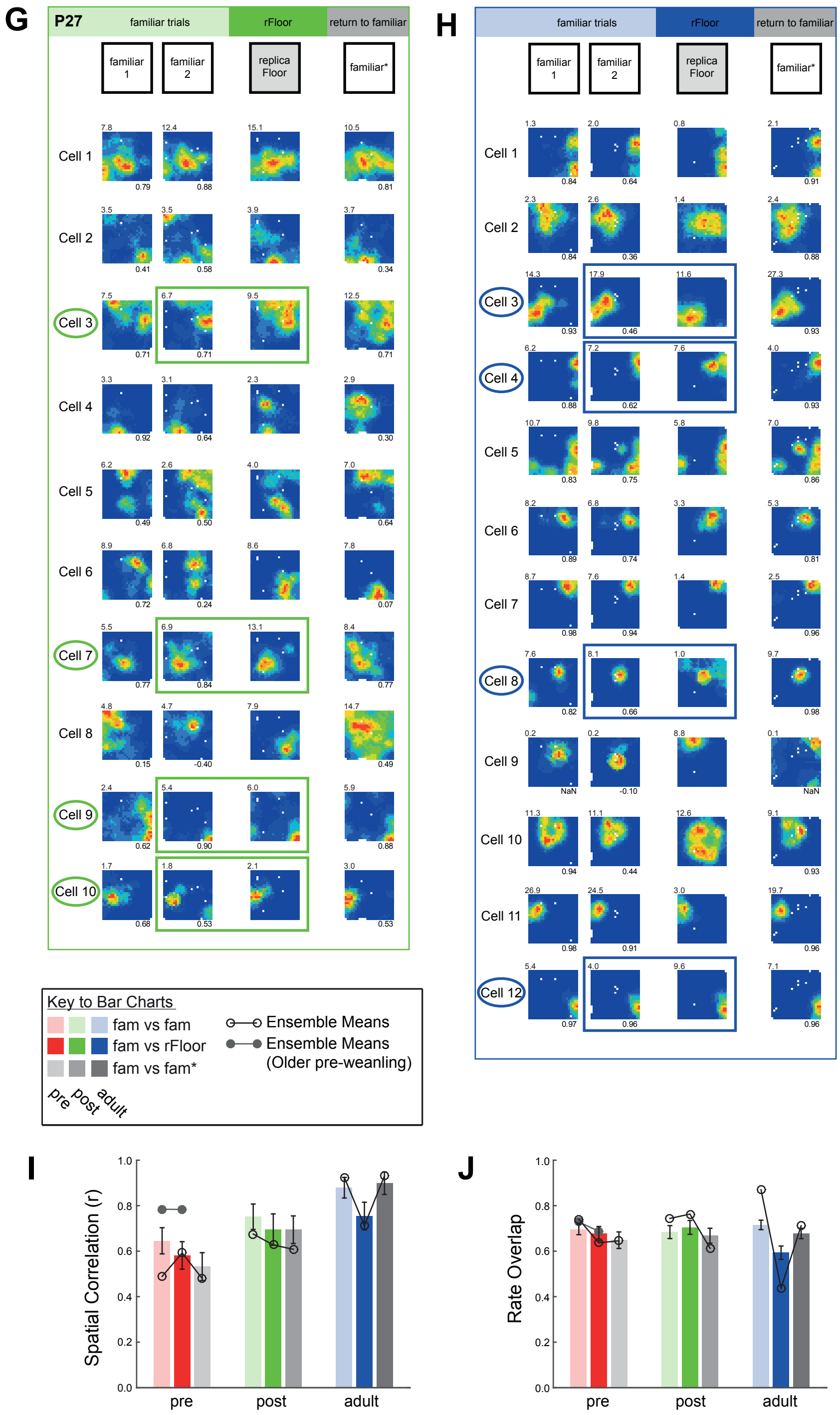

Supplemental Figure 3, continued. (**F-J**) 'rFloor' condition, where only the floor of the environment was replaced with a visually identical replica.

(**F-H**) Complete ensembles of simultaneously recorded place cells from which examples in main Figure 4D are drawn: (**A**), pre-weanling rats (two separate ensembles, from a younger and an older animal); (**B**), post-weanling rats and (**C**), adult animals. The cells shown in main figure 4D are highlighted with a circle around the cell number, to the left of the rate maps. Note that an additional trial in the familiar environment following the exposure to 'rFloor' is also shown, whenever available. The '//' symbol between familiar trials indicates that another environmental manipulation (not shown in this figure) was run in between the sessions shown. The numbers on bottom right of rate maps indicate spatial correlation (r) with the following trial, except for 'familiar*' trials where the correlation with the preceding familiar trial is indicated.

(**I, J**) Overall age group means (±SEM) for spatial correlation (**I**) and rate overlap (**J**). These panels show the same data as Figure 4E, 4F, but additionally show: the mean values of spatial correlation and rate overlap for the ensembles shown in S4F-H (black and grey lines and circles), and the overall mean (±SEM) comparisons between the familiar trials preceding and following the environmental manipulation (grey bars; 'return-to-familiar'). There are no significant differences within each age group between familiar and return-to-familiar spatial correlation (ANOVA: Trial, $F_{1,373}$=1.80, p=0.18; Trial x Age, $F_{2,373}$=3.22, p=0.04, but all post-hoc comparisons of familiar versus return-to-familiar within age group are n.s.: $SME_{(pre)}$, p=0.07, $SME_{(post)}$, p=0.20, $SME_{(ad)}$, p=0.17), or rate overlap (ANOVA: Trial, $F_{1,379}$=2.24, p=0.14; Trial x Age, $F_{2,379}$=0.15, p=0.86).
