# Supplementary material for: Place Cell Networks in Pre-weanling Rats Show Associative Memory Properties from the Onset of Exploratory Behavior
Source: Cereb Cortex. 2016 Jul 25;26(8):3627–36. doi: 10.1093/cercor/bhw174 (PMC4961032; doi:10.1093/cercor/bhw174)
Supplement: Supplementary Data [file supp_bhw174_bhw174supp_table4.pdf]

Supplemental Table 4

A

Figure/Panel	Environment	Measure	Statistical Test	Effect	Statistic	p	Post-Hoc			
1B	novel	SC	2x3-ANOVA	Age	$F_{2,496}$=42.01	<0.001		Pre	Post	Adult
				Environment	$F_{1,496}$=533.6	<0.001	SME(Env)	<0.001	<0.001	<0.001
				Interaction	$F_{2,496}$=27.49	<0.001				
1B inset	novel	SC	T-Test	Environment	t(91)=8.35	<0.001				
1D	novel	RO	2x3-ANOVA	Age	$F_{2,535}$=1.85	0.16		Pre	Post	Adult
				Environment	$F_{1,535}$=130.4	<0.001	SME(Env)	<0.001	<0.001	<0.001
				Interaction	$F_{2,535}$=5.5	0.004				
1D inset	novel	RO	T-Test	Environment	t(101)=2.56	0.012				
								Fam vs Nov	Nov vs Nov	
1F (Pre-wean)	novel-novel	SC	3x1-ANOVA	Environment	$F_{(2,298)}$=70	<0.001	Tukey HSD (Env)	<0.001	0.003	
1F (Post-wean)			3x1-ANOVA	Environment	$F_{(2,127)}$=33	<0.001		<0.001	0.32	
1G (Pre-wean)		RO	3x1-ANOVA	Environment	$F_{(2,332)}$=15	<0.001	Tukey HSD (Env)	<0.001	0.016	
1G (Post-wean)			3x1-ANOVA	Environment	$F_{(2,139)}$=12	<0.001		<0.001	0.51	
2B	rEnv	SC	2x3-ANOVA	Age	$F_{2,420}$=90.64	<0.001		Pre	Post	Adult
				Environment	$F_{1,420}$=65.4	<0.001	SME(Env)	<0.001	0.033	<0.001
				Interaction	$F_{2,420}$=3.19	0.042				
2B inset	rEnv	SC	T-Test	Environment	t(79)=4.78	<0.001				
2D	rEnv	RO	2x3-ANOVA	Age	$F_{2,421}$=2.79	0.062		Pre	Post	Adult
				Environment	$F_{1,421}$=4.83	0.028	SME(Env)	<0.001	0.15	0.012
				Interaction	$F_{2,421}$=5.73	0.003				
2D inset	rEnv	RO	T-Test	Environment	t(79)=1.06	0.29				
3B, left	rWalls	SC	2x3-ANOVA	Age	$F_{2,227}$=52.55	<0.001		Pre	Post	Adult
				Environment	$F_{1,227}$=0.39	0.53	SME(Env)	n/a	n/a	n/a
				Interaction	$F_{2,227}$=0.94	0.39				
3B, left inset	rWalls	SC	T-Test	Environment	t(10)=-0.39	0.7				
3C, left	rWalls	RO	2x3-ANOVA	Age	$F_{2,227}$=0.24	0.79		Pre	Post	Adult
				Environment	$F_{1,227}$=0.76	0.39	SME(Env)	n/a	n/a	n/a
				Interaction	$F_{2,227}$=0.68	0.51				
3C, left inset	rWalls	RO	T-Test	Environment	t(10)=0.87	0.4				
3E, left	rFloor	SC	2x3-ANOVA	Age	$F_{2,400}$= 27.47	<0.001		Pre	Post	Adult
				Environment	$F_{1,400}$= 13.68	<0.001	SME(Env)	0.3	0.26	0.001
				Interaction	$F_{2,400}$=3.43	0.033				
3E, left inset	rFloor	SC	T-Test	Environment	t(52)=-0.34	0.74				
3F, left	rFloor	RO	2x3-ANOVA	Age	$F_{2,406}$=1.26	0.29		Pre	Post	Adult
				Environment	$F_{1,406}$=2.88	0.09	SME(Env)	0.67	0.65	<0.001
				Interaction	$F_{2,406}$=3.8	0.023				
3F, left inset	rFloor	RO	T-Test	Environment	t(52)=0.23	0.82				
								Fam vs rWalls	Fam vs rFloor	Fam vs rEnv
3G	rW, rF, rEnv	SC	4x1-ANOVA	Environment	$F_{3,319}$=31.06	<0.001	Tukey HSD (Env)	0.9	0.99	<0.001
3G inset	rW, rF, rEnv	SC	4x1-ANOVA	Environment	$F_{3,128}$=7.86	<0.001		0.92	1	<0.001
3H	rW, rF, rEnv	RO	4x1-ANOVA	Environment	$F_{3,322}$=6.04	0.001		1	0.78	<0.001

B

	pre-wean		post-wean		adult	
**Spatial Correlation**	t-statistic	p	t-statistic	p	t-statistic	p
Novel	t(108)=14.3	<0.001	t(45)=8.1	<0.001	t(66)=17.3	<0.001
rEnv	t(74)=8.3	<0.001	t(49)=2.3	0.03	t(76)=6.2	<0.001
rWalls	t(44)=-1.6	0.12	t(33)=0.9	0.38	t(34)=-0.46	0.65
rFloor	t(54)=1.3	0.22	t(49)=1.5	0.14	t(89)=5.4	<0.001
**Rate Overlap**						
Novel	t(118)=4.8	<0.001	t(54)=3.5	<0.001	t(68)=9.2	<0.001
rEnv	t(74)=3.7	<0.001	t(49)=-1.6	0.13	t(76)=1.6	0.11
rWalls	t(44)=0.2	0.87	t(33)=-1.6	0.11	t(34)=-0.5	0.6
rFloor	t(57)=0.6	0.57	t(49)=-1.2	0.23	t(91)=3.6	<0.001

C

Figure/Panel	Environment	Measure	Statistical Test	pre-wean		post-wean			
				statistic	p	statistic	p	statistic	p
1C	novel	SC	2-way KS-test	0.69	<0.001	0.73	<0.001	0.85	<0.001
1E	novel	RO	2-way KS-test	0.37	<0.001	0.45	<0.001	0.65	<0.001
2C	rEnv	SC	2-way KS-test	0.51	<0.001	0.19	0.29	0.37	<0.001
2E	rEnv	RO	2-way KS-test	0.37	<0.001	0.17	0.41	0.24	0.012
3B, right	rWalls	SC	2-way KS-test	0.22	0.19	0.21	0.39	0.23	0.24
3C, right	rWalls	RO	2-way KS-test	0.13	0.79	0.18	0.61	0.15	0.8
3D, right	rFloor	SC	2-way KS-test	0.14	0.59	0.16	0.52	0.35	<0.001
3E, right	rFloor	RO	2-way KS-test	0.16	0.45	0.11	0.9	0.25	0.003

Supplemental Table 4. Full statistical results, and numbers of cells recorded, for Figures 1-3 (**A**) Results for all parametric tests (ANOVAs and t-tests). The table shows detailed results of statistical analyses for all panels in Figures 1-3 in which data is plotted as mean±SEM. Factorial design of analysis (ANOVAs) is indicated in the 'Statistical Test' column. All main effects and interactions are indicated with respective F or t and p values. Simple Main Effects (SME) were used as post-hoc analyses for 2-way ANOVAs, Tukey's Honest Significant Difference (Tukey HSD) was used as post-hoc analysis for 1-way ANOVAs. (**B**) Statistical analysis of same data as in (A) using paired sample t-tests, instead of ANOVAs. (**C**) Results for non-parametric tests comparing cumulative distribution functions (CDFs) for spatial correlation and rate overlap scores (Kolmogorov–Smirnov [KS] tests). Test statistics and p-values are indicated for all age groups and represent comparisons between average familiar trials (calculated across trials before any manipulations were run) and familiar vs environmental manipulations. p<0.05 are shown in red.

D

	Novel				
	mean age	N animals	N Sessions (1st exposure, Repeat exposure)	mean ensemble size	N Cells
Pre-weanling	18.9±1.4	11	17 (11, 6)	8.4±4.8	142
Post-weanling	23.6±2.5	9	10 (9, 1)	6.4±2.5	64
Adult	n/a	7	9 (7, 2)	10.2±5.4	92
	rEnv				
	mean age	N animals	N Sessions (1st exposure, Repeat exposure)	mean ensemble size	N Cells
Pre-weanling	18.4±1.5	13	20 (13, 7)	4.1±3.4	81
Post-weanling	25.9±2.4	7	10 (7, 3)	5.2±2.9	52
Adult	n/a	7	7 (7, 0)	13.1±3.8	92

	rWalls				
	mean age	N animals	N Sessions (1st exposure, Repeat exposure)	mean ensemble size	N Cells
Pre-weanling	19.4±1.2	6	7 (6, 1)	6.4±4	45
Post-weanling	26.1±2.8	5	7 (5, 2)	5±2.1	35
Adult	n/a	3	3 (3, 0)	13±6.1	39
	rFloor				
	mean age	N animals	N Sessions (1st exposure, Repeat exposure)	mean ensemble size	N Cells
Pre-weanling	18.5±1.7	12	15 (12, 3)	3.9±3.4	58
Post-weanling	25.9±2.3	6	9 (6, 3)	5.9±2.8	53
Adult	n/a	8	9 (8, 1)	11.2±4.1	101

Supplemental Table 4, continued

(**D**) Mean age (mean±SD), number of animals, number of recording sessions (showing also the number of first exposures for each animal and the number of repeat exposures), mean size of place cell ensembles (mean±SD) and numbers of total place cells recorded, for all environmental manipulations. The environmental manipulations used in this study included both the first exposure to each manipulation for each rat, as well as repeat exposures (see third column above). To confirm that there was no effect of the amount of experience in the manipulated environments, for the two conditions in which remapping occurred in young rats ('novel' and 'rEnv') the remapping scores were divided by whether they were obtained from a 1st exposure or repeat exposure, and a 2x2 ANOVA (Exposure: 1st, repeat; Age: pre-weanling, post-weanling) was run for each score and for each manipulation. For all ANOVAs, neither the Exposure main effect nor the Age*Exposure interaction were significant. (Novel, SC: Exp, $F(1,165)=0.57$, $p=0.45$; Exp*Age, $F(1,165)=0.73$, $p=0.39$. Novel, RO: Exp, $F(1,196)=2.16$, $p=0.14$; Exp*Age, $F(1,196)=0.24$, $p=0.62$. rEnv, SC: Exp, $F(1,126)=1.76$, $p=0.19$; Exp*Age, $F(1,126)=0.08$, $p=0.77$. rEnv, RO: Exp, $F(1,126)=0.14$, $p=0.70$; Exp*Age, $F(1,126)=0.29$, $p=0.59$.
