# Supplementary material for: Place Cell Networks in Pre-weanling Rats Show Associative Memory Properties from the Onset of Exploratory Behavior
Source: Cereb Cortex. 2016 Jul 25;26(8):3627–36. doi: 10.1093/cercor/bhw174 (PMC4961032; doi:10.1093/cercor/bhw174)
Supplement: Supplementary Data [file supp_bhw174_bhw174supp_fig5.pdf]

Supplemental Figure 5

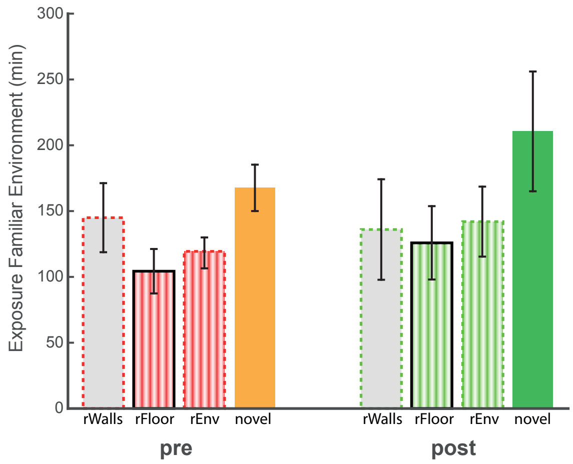

Supplemental Figure 5. Average exposure of animals to familiar environment before environmental manipulations were conducted does not differ between age groups and manipulation type. Data represents the average time spent in the familiar environment (mean±SEM) before environmental manipulations were conducted. Data is shown only for pre- and post-weanling pups.

There is neither a significant difference in exposure times between age groups nor a systematic difference across environmental manipulations (ANOVA: Age x Manipulation Type: Age, $F_{1,87}=1.19$, $p=0.28$; Manipulation Type, $F_{3,87}=3.68$, $p=0.015$; Age x Manipulation Type, $F_{3,87}=0.3$, $p=0.83$; post-hoc tests for Manipulation Type [Tukey HSD], all $p>0.7$, except ‘rFloor’ vs Novel, $p=0.015$; ‘rEnv’ vs Novel, $p=0.05$).
