# Supplementary material for: Place Cell Networks in Pre-weanling Rats Show Associative Memory Properties from the Onset of Exploratory Behavior
Source: Cereb Cortex. 2016 Jul 25;26(8):3627–36. doi: 10.1093/cercor/bhw174 (PMC4961032; doi:10.1093/cercor/bhw174)
Supplement: Supplementary Data [file supp_bhw174_bhw174supp_fig6.pdf]

Supplemental Figure 6

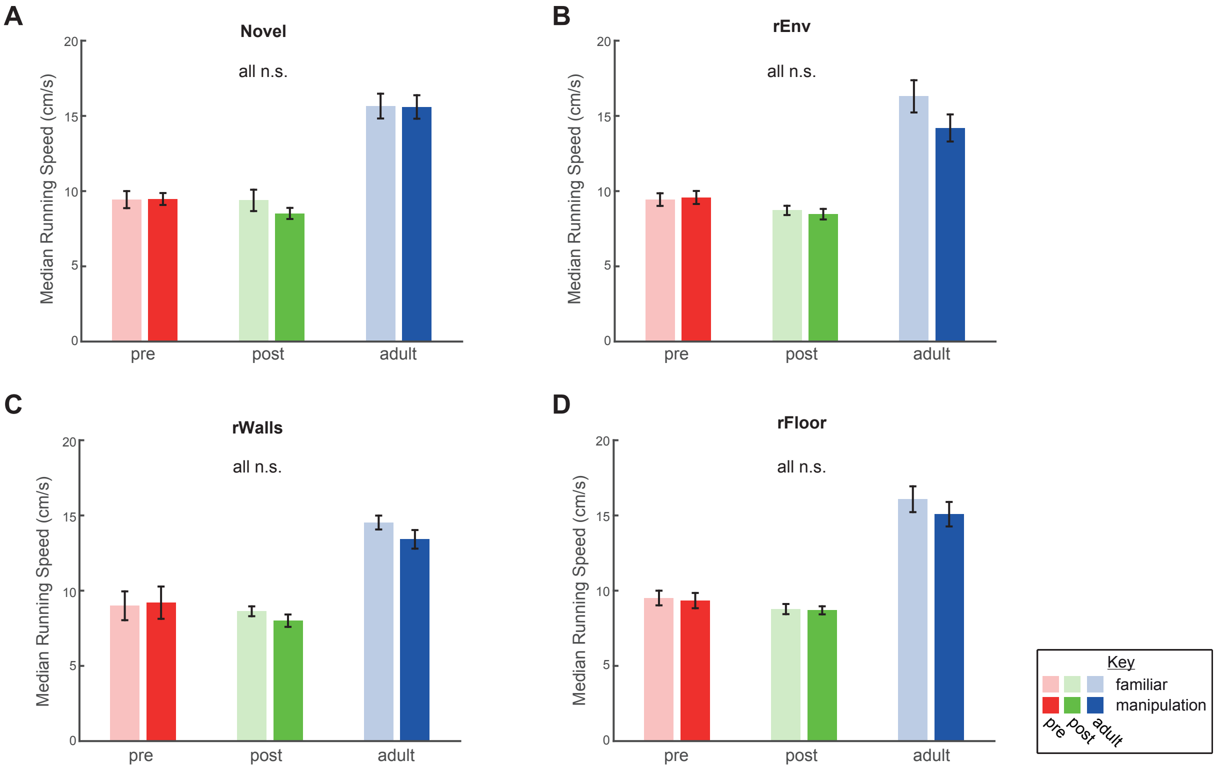

Supplemental Figure 6. Average median running speed of animals does not vary between familiar trials and environmental manipulations. Data represents average median running speeds (mean±SEM) across familiar trials (pale colours) as well as manipulation trials (bold colours).

(**A**) Familiar vs. Novel environment (ANOVA, Age x Trial Type: Age, $F_{2,66}$=64.6, p<0.001; Trial, $F_{1,66}$=0.34, p=0.56; Age x Trial, $F_{2,66}$=0.33, p=0.72). (**B**) Familiar vs. 'rEnv' (ANOVA, Age x Trial Type: Age, $F_{2,68}$=60.4, p<0.001; Trial, $F_{1,68}$=2.36, p=0.13; Age x Trial, $F_{2,68}$=1.87, p=0.16). (**C**) Familiar vs. 'rWalls' (ANOVA, Age x Trial Type: Age, $F_{2,28}$=19.35, p<0.001; Trial, $F_{1,28}$=0.52, p=0.48; Age x Trial, $F_{2,28}$=0.31, p=0.74). (**D**) Familiar vs. 'rFloor' (ANOVA, Age x Trial Type: Age, $F_{2,60}$=74.9, p<0.001; Trial, $F_{1,60}$=0.74, p=0.39; Age x Trial, $F_{2,60}$=0.34, p=0.72).
