# Supplementary material for: Place Cell Networks in Pre-weanling Rats Show Associative Memory Properties from the Onset of Exploratory Behavior
Source: Cereb Cortex. 2016 Jul 25;26(8):3627–36. doi: 10.1093/cercor/bhw174 (PMC4961032; doi:10.1093/cercor/bhw174)
Supplement: Supplementary Data [file supp_bhw174_bhw174supp_fig7.pdf]

Supplemental Figure 7

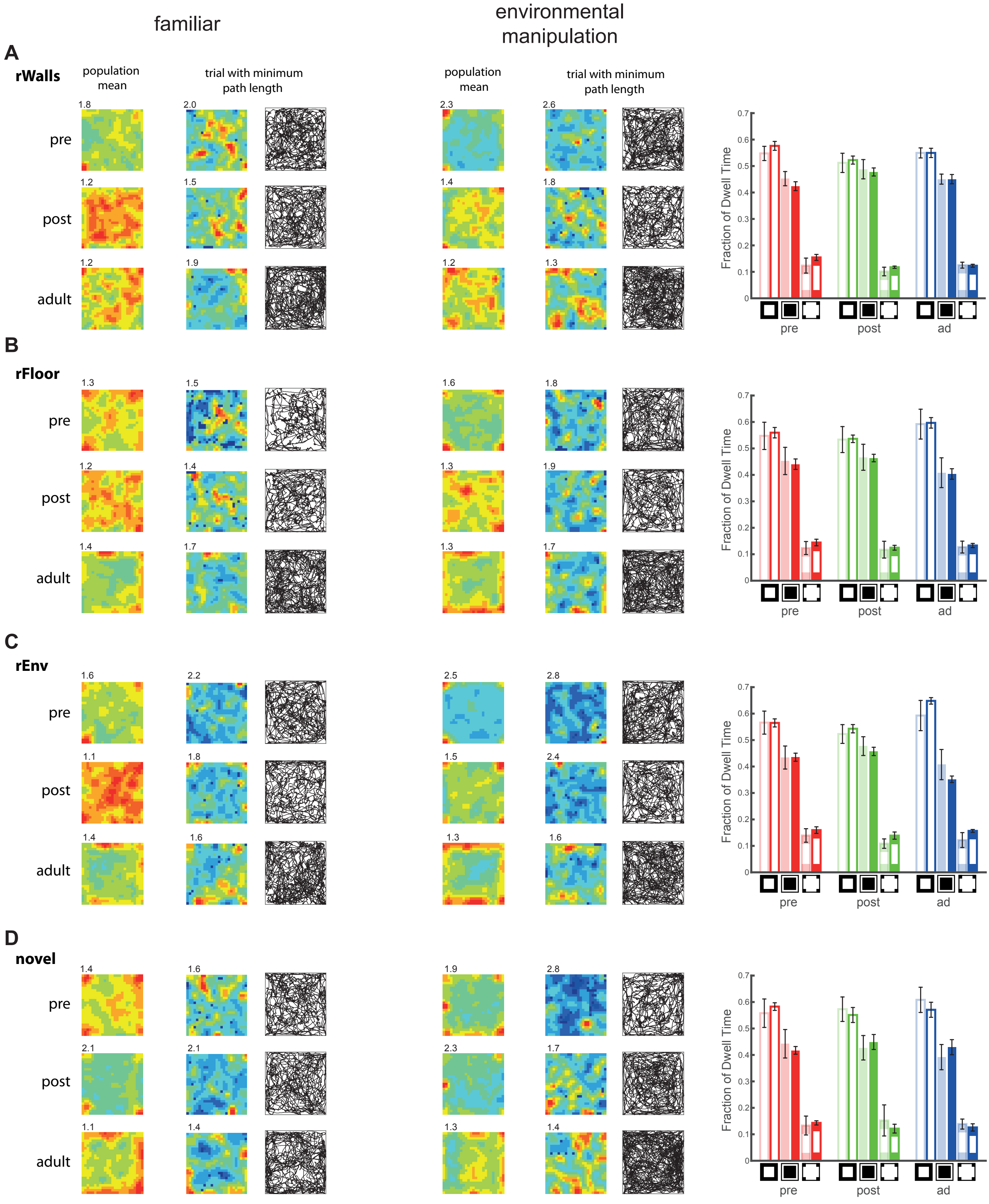

Supplemental Figure 7. Position sampling of environment does not vary systematically between familiar trials and environmental manipulations.

Each row represents, from left to right, the mean position map of all familiar trials before the environmental manipulation and the position map and path of the familiar trial with the shortest path length. The remaining three columns show the same data for the environmental manipulation itself. Position maps are shown as false-colour heat maps and are constructed correspondingly to rate maps. Bar charts depict mean±SEM fractions of dwell time in three zones (schematic below bars indicates respective zone highlighted in black) of the environment (edge: all bins with distance <10cm from nearest wall; centre: all bins with distance >10cm from nearest wall; corner: all bins with distance <10cm from the two nearest walls) for all three age groups (pre, post, ad). Data is shown for both familiar (pale colours) and environmental manipulations (bold colours). (**A-D**) Data for ‘rWalls’, ‘rFloor’, ‘rEnv’ and ‘Novel’ manipulation, respectively. For all ANOVAs (Trial x Zone for each age group and environmental manupulation there is a main effect of Zone (all p<0.001) but no effect Trial (all p>0.33). All interaction effects are n.s. (p>0.12), except for pre-weaners and ‘rWalls’ (p=0.046; all $SME_{(Zone)}$ n.s.) and ‘rEnv’ and adults (p=0.002; $SME_{(Edge)}/SME_{(Centre)}$=0.014).
