# Supplementary material for: Place Cell Networks in Pre-weanling Rats Show Associative Memory Properties from the Onset of Exploratory Behavior
Source: Cereb Cortex. 2016 Jul 25;26(8):3627–36. doi: 10.1093/cercor/bhw174 (PMC4961032; doi:10.1093/cercor/bhw174)
Supplement: Supplementary Data [file supp_bhw174_bhw174supp_fig8.pdf]

Supplemental Figure 8

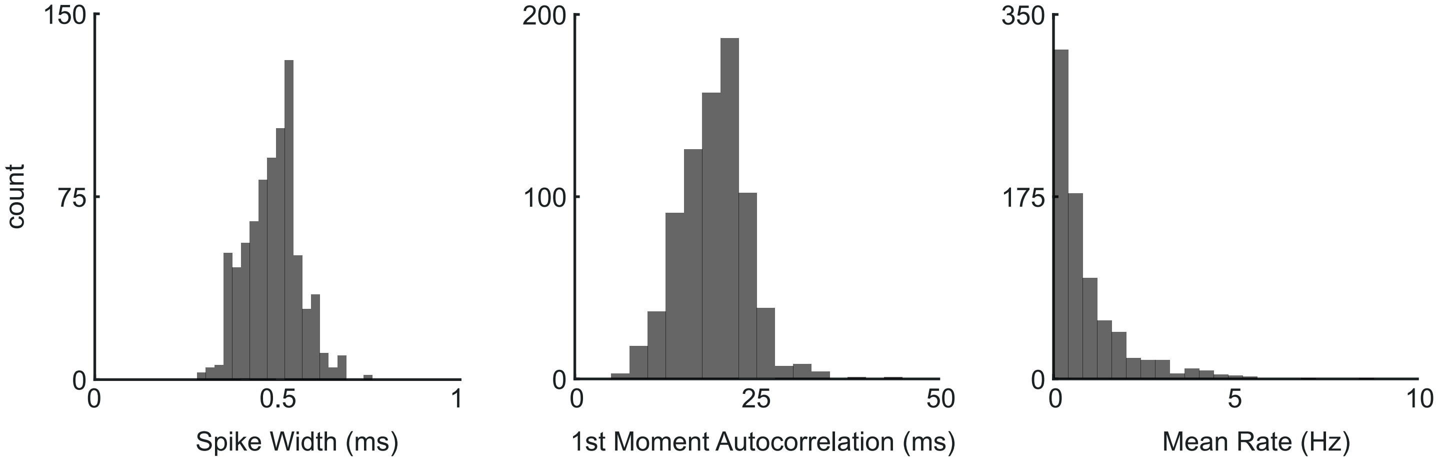

Supplemental Figure 8. Properties of complex spike cells. Shown are distributions for all three parameters that were used for k-means clustering to separate interneurons and pyramidal cells. Only data for complex spike cells recorded from rat pups is shown.

Shown are the distributions of putative complex spike cell properties for spike duration (left), mean of the autocorrelation (across 50ms; middle) and average firing rate (right).
