# Supplementary material for: Place Cell Networks in Pre-weanling Rats Show Associative Memory Properties from the Onset of Exploratory Behavior
Source: Cereb Cortex. 2016 Jul 25;26(8):3627–36. doi: 10.1093/cercor/bhw174 (PMC4961032; doi:10.1093/cercor/bhw174)
Supplement: Supplementary Data [file supp_bhw174_bhw174supp_fig9.pdf]

Supplemental Figure 9

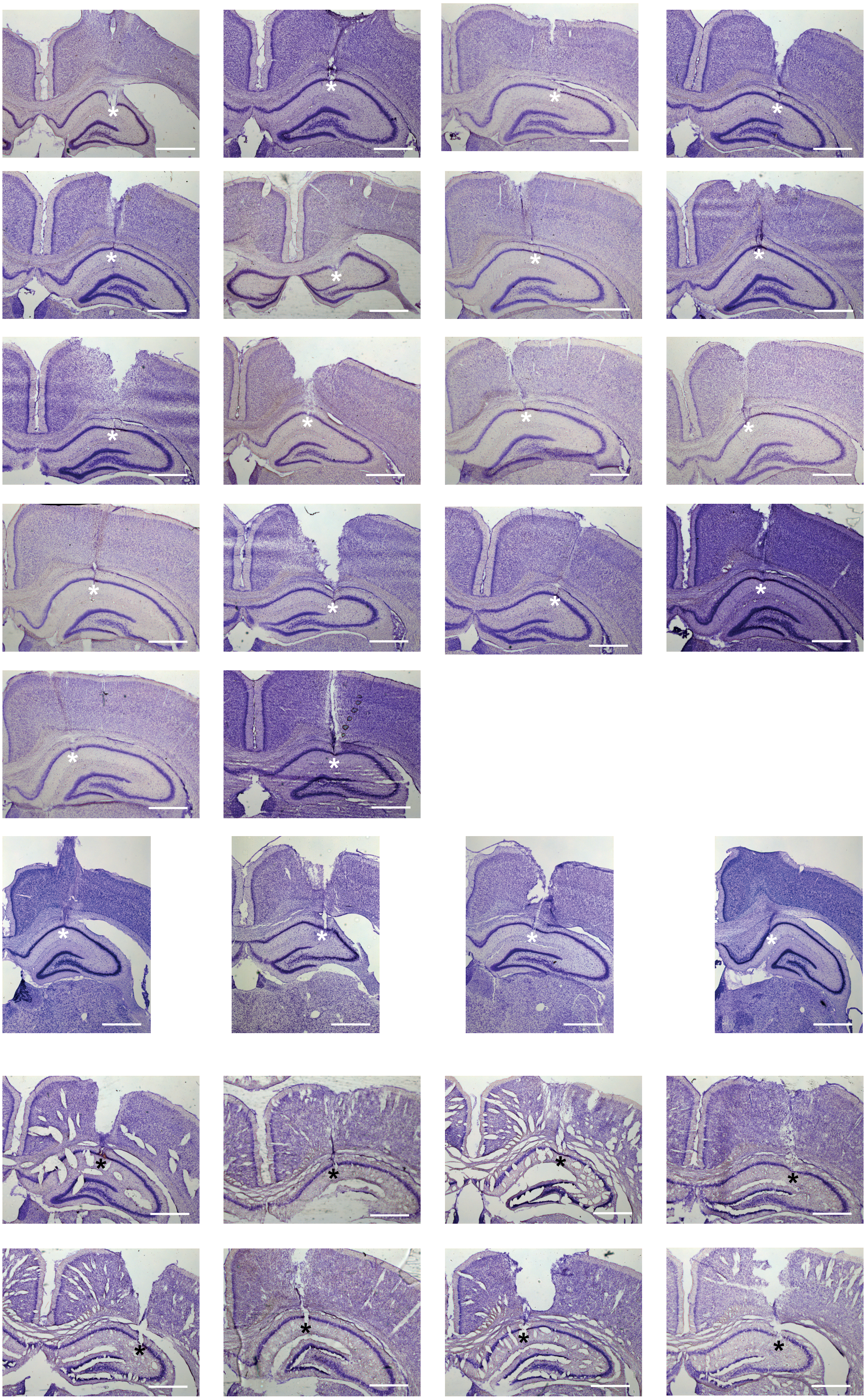

Supplemental Figure 9. Confirmation of electrode positions by post mortem Nissl stainings.

Shown are histological sections (30µm) through the dorsal hippocampus of brains from rat pups used in this study. Scale bar indicates 1mm and asterisk indicates position where hippocampal layer was penetrated by electrodes. Sections were stained with Cresyl violet (Nissl staining). Eight brains (bottom two rows) show cryo-damage resulting from a storage fridge malfunction, which occurred before brains had been cryo-protected. Two further brains were damaged to such an extent as to prevent electrode tract localisation; these brains are omitted from the figure.
